# Coronavirus Antibody Responses before COVID-19 Pandemic, Africa and Thailand

**DOI:** 10.3201/eid2811.221041

**Published:** 2022-11

**Authors:** Yifan Li, Mélanie Merbah, Suzanne Wollen-Roberts, Bradley Beckman, Thembi Mdluli, Isabella Swafford, Sandra V. Mayer, Jocelyn King, Courtney Corbitt, Jeffrey R. Currier, Heather Liu, Allahna Esber, Suteeraporn Pinyakorn, Ajay Parikh, Leilani V. Francisco, Nittaya Phanuphak, Jonah Maswai, John Owuoth, Hannah Kibuuka, Michael Iroezindu, Emmanuel Bahemana, Sandhya Vasan, Julie A. Ake, Kayvon Modjarrad, Gregory Gromowski, Dominic Paquin-Proulx, Morgane Rolland

**Affiliations:** Walter Reed Army Institute of Research, Silver Spring, Maryland, USA (Y. Li, M. Merbah, S. Wollen-Roberts, B. Beckman, T. Mdluli, I. Swafford, S.V. Mayer, J. King, C. Corbitt, H. Liu, A. Esber, S. Pinyakorn, A. Parikh, L.V. Francisco, S. Vasan, J.A. Ake, K. Modjarrad, G. Gromowski, D. Paquin-Proulx, M. Rolland);; Henry M. Jackson Foundation for the Advancement of Military Medicine, Inc., Bethesda, Maryland, USA (Y. Li, M. Merbah, S. Wollen-Roberts, B. Beckman, T. Mdluli, I. Swafford, H. Liu, A. Esber, S. Pinyakorn, A. Parikh, L.V. Francisco, S. Vasan, D. Paquin-Proulx, M. Rolland);; Institute of HIV Research and Innovation, Bangkok, Thailand (N. Phanuphak);; HJF Medical Research International, Kericho, Kenya (J. Maswai, J. Owuoth, M. Iroezindu, E. Bahemana);; Makerere University Walter Reed Project, Kampala, Uganda (H. Kibuuka)

**Keywords:** COVID-19, viruses, respiratory infections, severe acute respiratory syndrome coronavirus 2, SARS-CoV-2, SARS, coronavirus disease, zoonoses, coronavirus, serosurvey, HIV-1, Africa, Thailand

## Abstract

Prior immune responses to coronaviruses might affect human SARS-CoV-2 response. We screened 2,565 serum and plasma samples collected from 2013 through early 2020, before the COVID-19 pandemic began, from 2,250 persons in 4 countries in Africa (Kenya, Nigeria, Tanzania, and Uganda) and in Thailand, including persons living with HIV-1. We detected IgG responses to SARS-CoV-2 spike (S) subunit 2 protein in 1.8% of participants. Profiling against 23 coronavirus antigens revealed that responses to S, subunit 2, or subunit 1 proteins were significantly more frequent than responses to the receptor-binding domain, S-Trimer, or nucleocapsid proteins (p<0.0001). We observed similar responses in persons with or without HIV-1. Among all coronavirus antigens tested, SARS-CoV-2, SARS-CoV-1, and Middle East respiratory syndrome coronavirus antibody responses were much higher in participants from Africa than in participants from Thailand (p<0.01). We noted less pronounced differences for endemic coronaviruses. Serosurveys could affect vaccine and monoclonal antibody distribution across global populations.

COVID-19 clinical manifestations range from asymptomatic infection to death. Whether prior immune responses to human coronaviruses affect responses to SARS-CoV-2 remains unclear. At the population level, disparities in COVID-19 outcomes have been observed across geographic regions. For instance, countries in Africa have reported lower mortality rates than high-income countries, which can be attributed to the small percentage of persons in the oldest age groups and to underreporting (*1*,*2*). Previous responses to endemic coronaviruses also could influence how different populations responded to SARS-CoV-2.

Findings conflict as to whether previous coronavirus antigen responses cross-react with SARS-CoV-2. Depending on the antigen and cohort tested, binding responses have been detected in prepandemic samples at varying frequencies, but neutralizing antibodies have been identified in fewer samples (*3*–*8*). Some studies of prepandemic samples indicated that neutralizing responses to endemic coronaviruses could protect against SARS-CoV-2 infection, but the effects of previous coronavirus responses on SARS-CoV-2 have not been clearly elucidated (*6*,*7*,*9*–*13*).

To investigate coronavirus-specific antibody responses in different settings, we analyzed 2,565 samples collected during 2013 through early 2020 from persons living with HIV-1 (PLHIV) and persons without HIV in Kenya, Nigeria, Tanzania, Uganda, and Thailand. We profiled antibody binding responses to coronavirus antigens, including spike (S) and nucleocapsid (N) proteins of SARS-CoV-2, SARS-CoV-1, MERS-CoV, and 4 endemic coronaviruses. We further evaluated a subset of samples with strong binding responses for neutralizing, antibody-dependent cellular phagocytosis (ADCP), and antibody-dependent cellular cytotoxicity (ADCC) responses. We compared responses across geographic locations and according to HIV-1 status.

## Methods

### Ethics Statement

We adhered to the policies for protection of human subjects, as prescribed in AR70-25 (*14*). All participants provided written informed consent. We used samples collected in 3 clinical cohort studies that investigated HIV-1 and other infectious diseases. Institutional review boards at local institutions and at Walter Reed Army Institute of Research approved the study (approval nos. WRAIR 1494, WRAIR 1897, and WRAIR 2383).

### Samples and Antigens

We obtained serum and plasma specimens from 2 study cohorts in Africa and 1 in Thailand. Cohorts in Africa included the RV329 African Cohort Study (RV329/AFRICOS), which predominantly enrolled PLHIV with chronic infection, and study RV466 of the Joint West Africa Research Group (RV466/JWARG), which was designed to diagnose acute febrile illnesses in Nigeria. The cohort in Thailand was from the RV254 South East Asia Research Collaboration in HIV (RV254/SEARCH 010) study, which enrolls persons with acute HIV-1 infection. For negative controls, we used prepandemic plasma samples, including Zika Negative Plasma (SeraCare, https://www.seracare.com), Pooled Normal Human Plasma (Innovative Research, https://www.innov-research.com), and 2 human serum coronavirus panels, MSRM-CR1 and HMSRM-CR22 (BioIVT, https://bioivt.com). For positive controls, we used 2 SARS-CoV-2–positive plasma samples with high neutralization titers and 2 serum panels, HMSRM-COVIDPOS and HMSRM-COVIDREC (BioIVT). We also used 12 matched SARS-CoV-2 patient convalescent serum and plasma samples (Innovative Research). We divided 51 antigens into custom panels, including panels for coronaviruses (SARS-CoV-2, SARS-CoV-1, MERS-CoV, OC43, NL63, HKU1, 229E), flaviviruses, and HIV-1 (Appendix Table 1). We included an alphavirus, chikungunya Envelope 1 antigen (E1), in the flavivirus panel.

### Bead-Based Multiplex Assay

We adapted assays from a previous study (*15*). Per 1 million beads, we coupled 10 µg of antigen for flavivirus proteins (*15*); 2.5 µg for coronavirus nucleocapsid (N) proteins; 5 µg for HIV-1 proteins; and 5 µg for coronavirus spike (S) proteins, including subunit 1 (S1), subunit 2 (S2), receptor-binding domain (RBD), and S-Trimer. We used 1,200 conjugated beads of each antigen per well and ran samples in triplicate at 2 dilutions, 1:100 and 1:400. We tagged biotinylated Fc gamma receptors (FcγR) FcγRIIa-H131, FcγRIIb, FcγRIIIa-F158, and FcγRIIIb-NA2 (Duke Human Vaccine Institute, https://dhvi.duke.edu) with a 1:4 molar ratio of Streptavidin-R-Phycoetherin (ProZyme-Agilent, https://www.agilent.com). We stored the tagged FcγR conjugated beads at 4°C and used within 24 hours of conjugation. We detected FcγR binding by using 20 μL of Streptavidin-R-Phycoethrerin–bound FcγR (3μg/mL). We acquired >100 beads/antigen/well on a FlexMap-3D (Luminex Corporation, https://www.luminexcorp.com) by using the xPONENT software (Luminex Corporation, https://www.luminexcorp.com) to measure the median fluorescence intensity (MFI). We assayed 3 plates per detection and used 4 negative and 4 positive controls per plate, 2 each of plasma and serum. We used a conservative cutoff by setting the positive threshold at 6 times the response for the highest negative control (*16*).

### Pseudovirus Neutralization Assay

We ran assays as previously described (*17*). We reported neutralization values as fold changes corresponding to the ratio of the 50% inhibitory dilution (ID_50_) for SARS-CoV-1 or SARS-CoV-2 over the ID_50_ for S glycoprotein of vesicular stomatitis virus.

### ADCP

We measured ADCP as previously described (*18*). We incubated biotinylated SARS-CoV-2, SARS-CoV-1, or MERS-CoV S protein with yellow-green neutravidin-fluorescent beads (Molecular Probes-Thermo Fisher Scientific, https://www.thermofisher.com) for 2 h (37°C). We incubated a 100-fold dilution of beads to protein (10 μL) for 2 h at 37°C along with 100 μL of 100-fold diluted plasma before adding THP-1 cells (MilliporeSigma, https://www.sigmaaldrich.com) at 25,000 cells per well. After a 19-h incubation, we fixed cells with 4% formaldehyde solution (Tousimis, https://www.tousimis.com) and evaluated fluorescence on an LSRII (BD Biosciences, https://www.bdbiosciences.com). We calculated the phagocytic score by multiplying the percentage of bead-positive cells by the geometric MFI and dividing by 10^4^.

### ADCC

We generated SARS-CoV-2 S-expressing CEM cells by transfection with linearized plasmid (pcDNA3.1) encoding codon-optimized SARS-CoV-2 S that matched wild-type SARS-CoV-2 (GenBank accession no. MN988713). We plated 100,000 wild-type S-CEM cells per well with 100 μL of 1:100 diluted plasma in round bottom 96-well plates and incubated for 30 min at 4°C. We washed cells and added 200,000 Jurkat-Lucia NFAT-CD16 cells (Invivogen, https://www.invivogen.com) to each well in 100 μL of Iscove’s Modified Dulbecco Medium (Gibco-Thermo Fisher Scientific, https://www.thermofisher.com) and 10% fetal bovine serum (MilliporeSigma). We then centrifuged cells for 1 min at low speed and cocultured for 24 h at 37°C. Then, we added 50 μL of QUANTI-Luc (Invivogen) to 20 μL of coculture supernatant and immediately measured luminescence on an EnVision 2104 Multilabel Plate Reader (PerkinElmer, https://www.perkinelmer.com).

### Statistical Analysis

We used R (R Foundation for Statistical Computing, https://www.r-project.org) to visualize data and perform statistical analyses by using the ggplot2, ComplexHeatmap, and ggpubr packages. We performed Wilcoxon rank-sum tests to compare responses across antigens and participant groups and Wilcoxon signed-rank tests to compare antigen responses between samples collected in 2019 and 2020 from Thailand. We used Spearman ρ to estimate correlations between variables, a false discovery rate to adjust p values for multiple testing, and McNemar test to compare the proportion of reactivity to different antigens.

## Results

### SARS-CoV-2 S2 IgG Reactivity 

We analyzed coronavirus-specific antibody responses by using 2,565 samples collected from 2,250 participants in 5 countries (Appendix Table 2). Among participants, 1,868 (83%) were PLHIV, most of whom received antiretroviral treatment; participants from Africa initiated treatment during chronic infection, and participants from Thailand initiated treatment during acute infection. Most (1,652/2,565; 64%) samples were from participants in Africa: 653 from Kenya, 366 from Nigeria, 234 from Tanzania, and 399 from Uganda. Samples were collected in Africa during August 2013–February 2020; samples from Thailand were collected during August 2019–April 2020. Among 913 samples from Thailand, 598 were from PLHIV, including 315 participants who had 2 samples.

**Figure 1:**
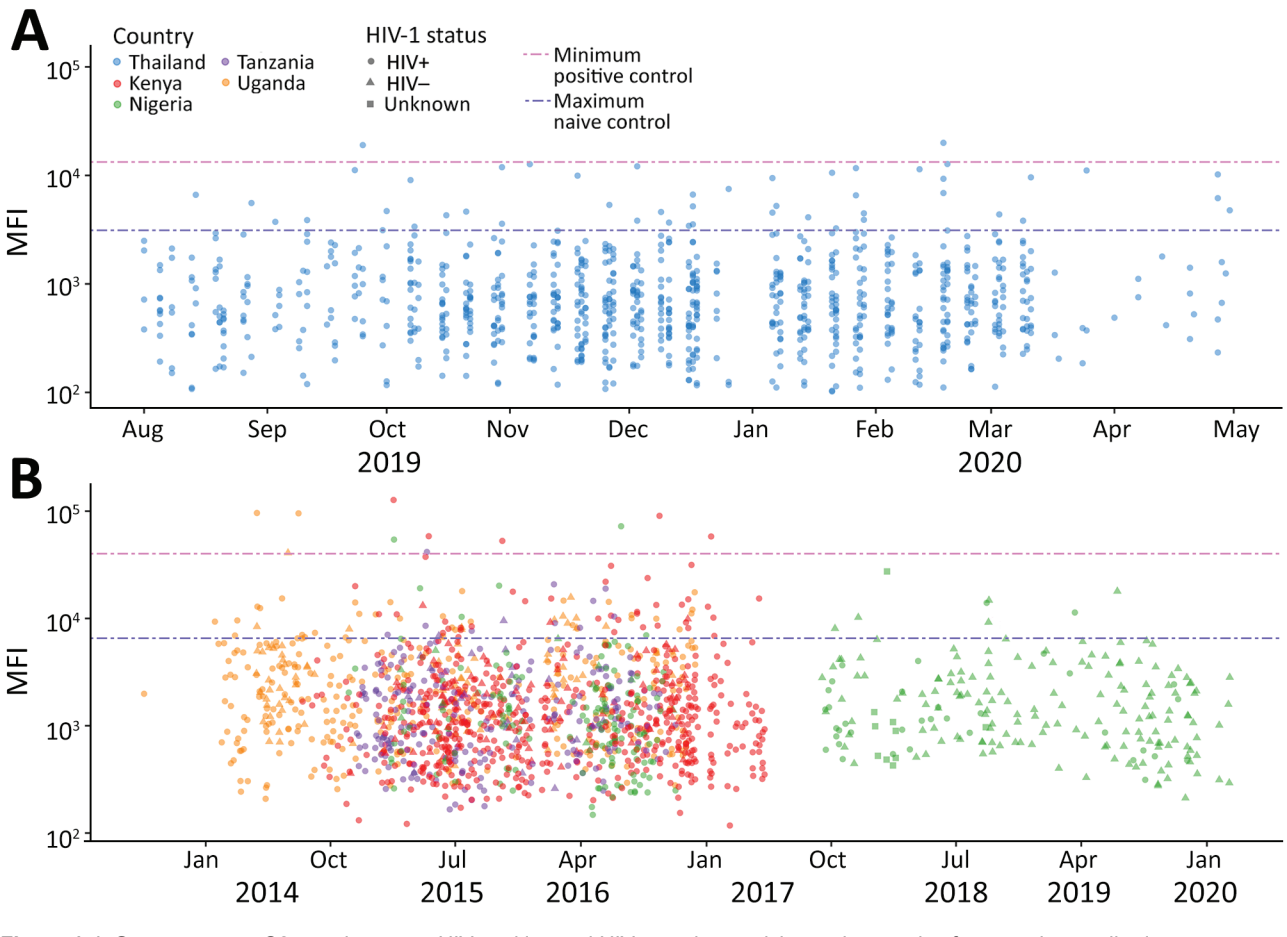
IgG responses to S2 protein among HIV-positive and HIV-negative participants in a study of coronavirus antibody responses before COVID-19 pandemic, Thailand (2013–2020) and Africa (2018–2020). A) Thailand; B) Kenya, Nigeria, Tanzania, and Uganda. We measured MFI for SARS-CoV-2 S2 IgG binding responses in 2,565 serum and plasma samples. Blue dashed line indicates maximum observed signal in 2 negative control samples; pink dashed line indicates minimum observed signal in positive control samples collected from SARS-CoV-2 convalescent patients. Symbols indicate the country of origin, collection date, and HIV-1 status of each participant. Dates indicate sample collection date. MFI, mean fluorescent intensity; S2, subunit 2 protein.

We screened all samples for IgG reactivity against the conserved S2 subunit of SARS-CoV-2 S protein (Figure 1). We selected for further analysis 173 samples that had a signal above the maximum seen with known negative samples: 108 from RV329/AFRICOS, 9 from RV466/JWARG, and 56 from RV254/SEARCH 010. Among samples from Africa, 33 (2% of all samples) had a signal-to-noise ratio (S/N) >6. Among the cohort from Thailand, 11 (1% of all samples) samples from 7 participants had S/N >6. Among 315 participants from Thailand, we detected no evidence of increased SARS-CoV-2 S2 IgG responses between samples collected in 2019 and those collected in 2020 (Appendix Figure 1). Overall, 1.78% of participants showed SARS-CoV-2–like S2 IgG responses before the pandemic, 5.38% when we considered S/N >3 as the cutoff. We noted no major differences across country of origin, sex, HIV-1 status, or year of sample collection; thus, we saw no evidence these samples corresponded to a specific subset of participants.

### Responses to Coronavirus Antigens

**Figure 2:**
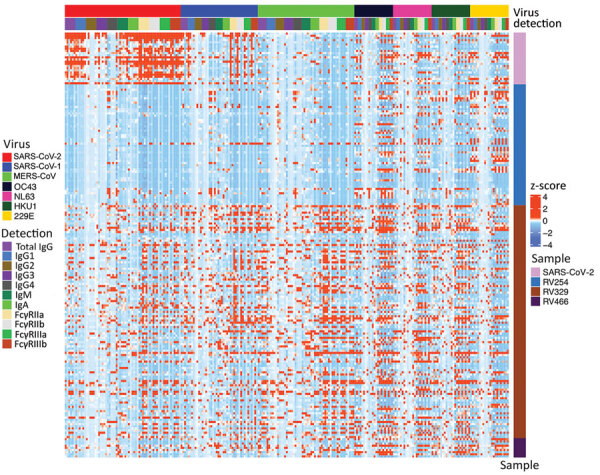
Heat map of coronavirus-specific antibody responses in a study of coronavirus antibody responses before COVID-19 pandemic, Thailand and Africa. We measured antibody responses for in 173 prepandemic serum and plasma samples and 12 samples collected from SARS-CoV-2 convalescent patients. Samples were tested for human coronaviruses SARS-CoV-2, SARS-CoV-1, MERS-CoV, OC43, NL63, HKU1, and 229E. Binding responses are given as z-scores. Each column corresponds to a specific antigen and detection combination. Each row represents a sample; the top 24 rows correspond to positive controls from SARS-CoV-2 convalescent patients. FcγR, Fc gamma receptor (FcγRIIa, FcγRIIb, FcγRIIIa, and FcγRIIIb).

**Figure 3:**
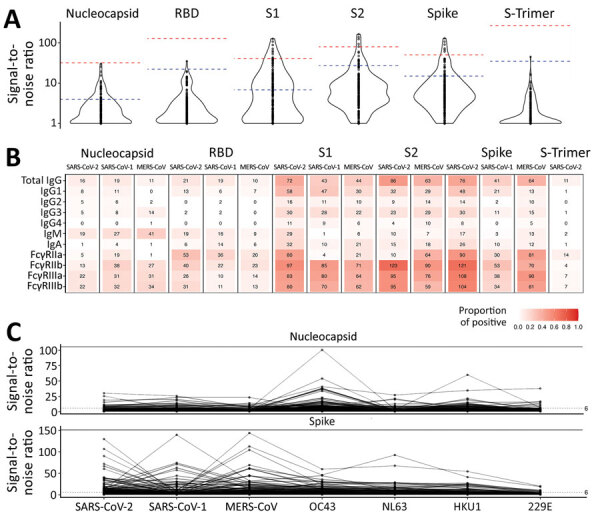
Comparison of antibody responses to human coronaviruses in serum and plasma samples collected before COVID-19 pandemic and from convalescent SARS-CoV-2 patients, Thailand and Africa. A) Violin plot comparing SARS-CoV-2 IgG binding responses against positive control samples. Blue dashed lines indicate median observed signal in positive control samples; pink dashed lines indicate maximum observed signal in positive control samples collected from SARS-CoV-2 convalescent patients. B) Number of coronavirus-positive samples detected by using a signal-to-noise ratio >6 across 3 outbreak coronaviruses and all antigens. C) IgG binding responses in nucleocapsid (top) and spike (bottom) proteins against all 7 human coronaviruses investigated. MERS-CoV, Middle East respiratory syndrome coronavirus; RBD, receptor-binding domain; S1, subunit 1; S2, subunit 2.

We tested the 173 selected samples by using a multiplex bead-based immunoassay to measure antibody responses against 23 human coronavirus antigens corresponding to S and N for all 7 human coronaviruses and for S1, S2, and RBD antigens for outbreak coronaviruses. We obtained 312,048 measurement that mapped isotypes, subclasses, and responses for FcγR-IIa, FcγR-IIb, FcγR-IIIa, and FcγR-IIIb (Figure 2). For SARS-CoV-2 antigens, 16 samples had IgG responses for N with S/N >6; for S antigens, 72 samples had S/N >6 for S1, 86 for S2, 21 for RBD, and 11 for S-Trimer (Figure 3, panels A, B). For all 2,250 cohort participants, these findings translate to SARS-CoV-2 reactivity ranging from 0.44% for S-Trimer to 3.69% for S2.

Compared with samples from 12 SARS-CoV-2 convalescent patients, 30 prepandemic samples showed higher SARS-CoV-2 responses for N and 28 were higher for S than the median observed across convalescent patients, but only 1 sample was above the median for RBD (Figure 3, panel A). No prepandemic samples showed RBD, S-Trimer, or N responses above the maximum signal seen for samples from convalescent patients; however, 5 to 9 prepandemic samples had S/N for S, subunit S2, and subunit S1 above the maximum seen in convalescent samples. We noted this pattern of lower recognition for N, RBD, or S-Trimer across all 3 outbreak coronaviruses; significantly fewer samples responded to N, RBD, or S-Trimer than to S, S1, or S2 (p<0.0001) (Figure 3, panel B). Using S/N >6, 76 samples showed IgG responses to S of SARS-CoV-2, 41 to S of SARS-CoV-1, and 64 to S of MERS-CoV; however, 16 samples showed IgG responses to N of SARS-CoV-2, 19 to N of SARS-CoV-1, and 11 to N of MERS-CoV. Few (15/76) samples with S responses also responded to RBD. Responses were more frequently detected against SARS-CoV-2 than SARS-CoV-1 (for S, p<0.0001) or MERS-CoV (for S and RBD, p<0.025). Across endemic coronaviruses, S and N of OC43 were recognized most frequently, albeit S was recognized less frequently (Figure 3, panel C). We noted strong positive relationships between IgG responses for SARS-CoV-2 and other coronaviruses. For S, Spearman correlations ranged from 0.58 for SARS-CoV-1 to 0.87 for MERS-CoV; for N, Spearman correlations ranged from 0.43 for 229E to 0.91 for SARS-CoV-1 (Appendix Figure 2). FcγR binding response rates were generally higher than those for Ig rates, but recognition patterns were similar, and far fewer persons’ samples recognized N (5–22 samples), RBD (26–53 samples), or S-Trimer (4–14 samples) than S (90–121 samples), S1 (80–97 samples), or S2 (10–123 samples) (p<0.0001) (Figure 3, panel B).

Samples from the Thailand cohort were collected during August 2019–April 2020, before documented SARS-CoV-2 infections in the cohort; 18 of 38 participants provided samples at 2 time points. For SARS-CoV-2 S2 IgG screening (Appendix Figure 1), we saw no evidence of increased SARS-CoV-2–specific reactivity in early 2020 compared with 2019 (Appendix Figure 3).

### Coronavirus-Specific Responses

**Figure 4:**
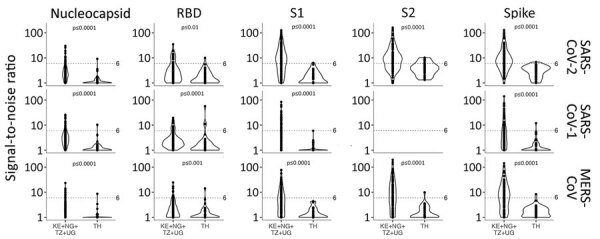
Violin plots of IgG signal-to-noise ratio comparing coronavirus antibody responses before COVID-19 pandemic, Thailand and Africa. We investigated IgG responses across 14 antigens from 3 coronaviruses, SARS-CoV-2, SARS-CoV-1, and Middle East respiratory syndrome coronavirus. Dotted line indicates signal-to-noise ratio cutoff. Significance was determined by Wilcoxon rank-sum test. KE, Kenya; NG, Nigeria; RBD, receptor-binding domain; S1, subunit 1; S2, subunit 2; TH, Thailand; TZ, Tanzania; UG, Uganda.

**Figure 5:**
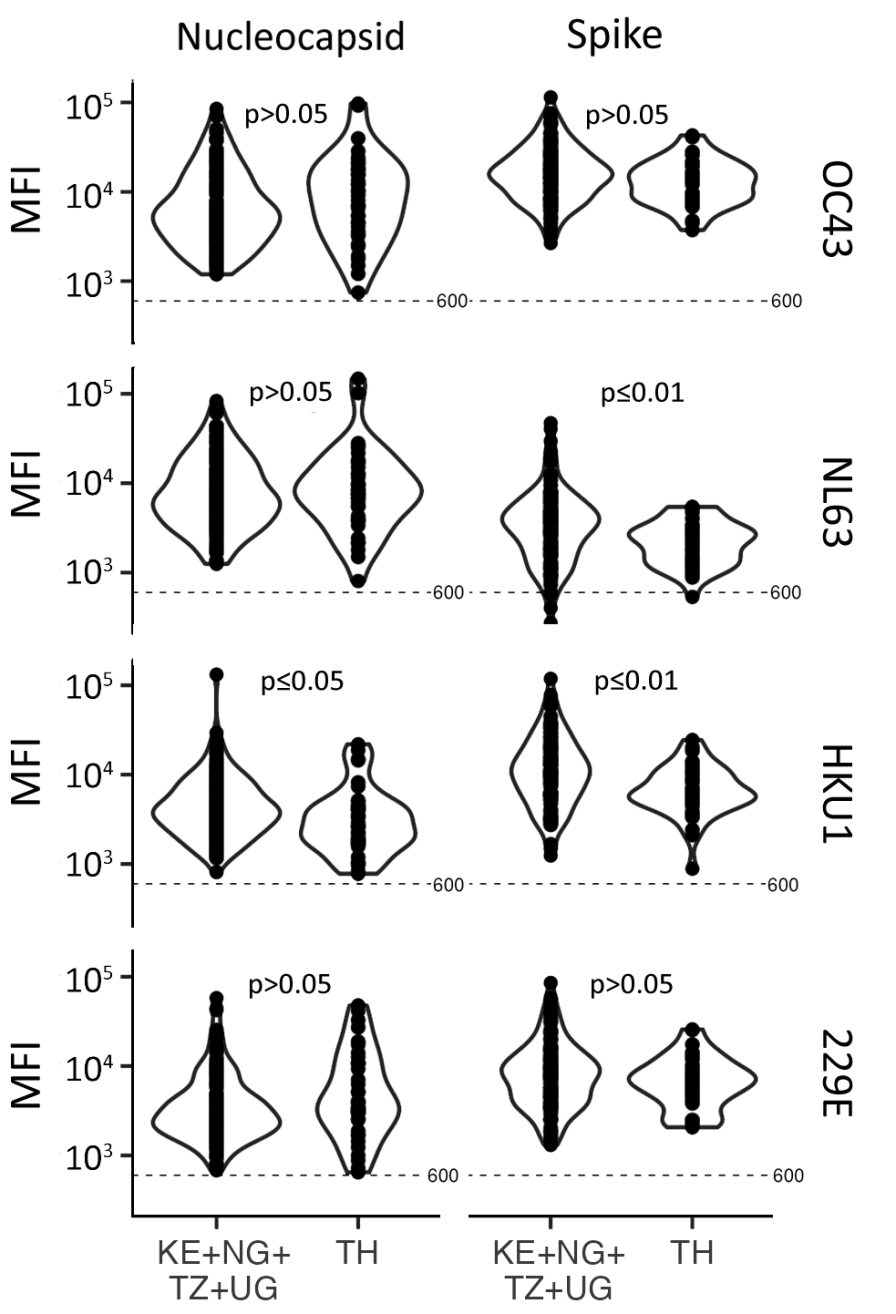
Violin plots of IgG mean fluorescent intensity for nucleocapsid and spike proteins of 4 endemic human coronaviruses in serum and plasma samples collected before the COVID-19 pandemic, Thailand and Africa. Samples comprised 117 participants from Kenya, Nigeria, Tanzania, and Uganda and 38 participants from Thailand.﻿ Significance was determined by Wilcoxon rank-sum test. Dotted line indicates MFI cutoff. KE, Kenya; MFI, mean fluorescent intensity; N, nucleocapsid; NG, Nigeria; RBD, receptor-binding domain; S1, subunit 1; S2, subunit 2; TH, Thailand; TZ, Tanzania; UG, Uganda.

**Figure 6:**
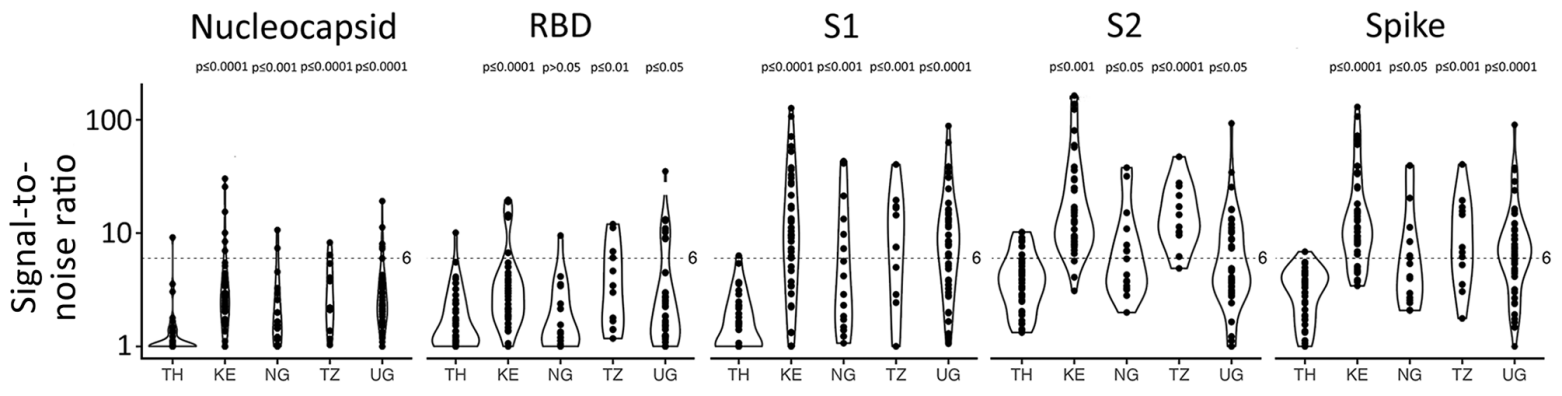
Violin plots of signal-to-noise ratio comparing SARS-CoV-2 IgG responses in serum and plasma samples before COVID-19 pandemic, Thailand and Africa. Dotted line indicates signal-to-noise ratio cutoff. Results show higher SARS-CoV-2 responses in participants from Africa than in participants from Thailand. Significance was determined by Wilcoxon rank-sum test. KE, Kenya; N, nucleocapsid; NG, Nigeria; RBD, receptor-binding domain; S1, subunit 1; S2, subunit 2; TH, Thailand; TZ, Tanzania; UG, Uganda.

We found a strikingly different pattern of reactivity in Kenya, Nigeria, Tanzania, and Uganda than in Thailand. Samples from participants in Africa had much higher SARS-CoV-2–like, SARS-CoV-1–like, and MERS-CoV–like responses (Figures 4–6). Although samples from Africa had more reactivity across all 7 coronaviruses than samples from Thailand, the difference was most striking for outbreak coronaviruses (Figure 4). Participants from Africa also had much higher SARS-CoV-2 IgG responses compared with participants from Thailand across all antigens except for S-Trimer (median S/N for S 7.95 vs. 3.4; p<0.01). We saw similar patterns for SARS-CoV-1 (median S/N for S 3.63 vs. 1.0; p<0.0001) and MERS-CoV (median S/N for S 7.0 vs. 1.64; p<0.0001). For endemic coronaviruses, responses tended to be higher in samples from Africa than in samples from Thailand but the difference was less pronounced: S responses for HKU1 and NL63 were significantly higher in participants from Africa (p<0.0037) but not for 229E or OC43 (p>0.097); however, N responses for HKU1 were significantly higher (p = 0.012) but not for OC43, NL63, and 229E (p>0.093) (Figure 5). We saw similar patterns for IgM and IgA responses (Appendix Figure 4). We noted more variability across samples from Africa than across those from Thailand. We tested whether this was because of the larger number of samples pooled from Africa by analyzing data from each country separately (Figure 6), or by downsampling data from each of the 4 countries (Appendix Figure 5). These comparisons showed lower coronavirus-specific responses in samples from Thailand than in samples from countries in Africa (Figure 6; Appendix Figure 5). Comparisons across the 4 countries in Africa showed different distributions, but we noted no consistent country-specific patterns across antigens or detection reagents (Appendix Figure 6).

### Correlation between Coronavirus and Other Pathogen Responses

**Figure 7:**
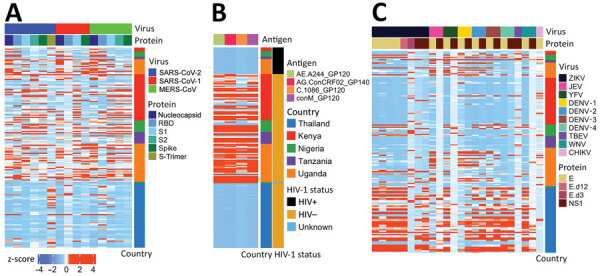
Heatmaps for outbreak coronaviruses, HIV-1, and flavivirus responses compared in a study of coronavirus antibody responses before COVID-19 pandemic, Thailand and Africa. A) IgG binding responses against SARS-CoV-2, SARS-CoV-1, and MERS-CoV. B) IgG binding responses against HIV-1 envelope antigens corresponding to CRF01_AE, CRF02_AG, subtype C, and group M. C) IgG binding responses against flaviviruses. Binding responses are presented as Z scores. Each column corresponds to a specific antigen. Each row represents a sample; the country of origin and HIV-1 status are marked in different colors. CHIKV, chikungunya virus; DENV, dengue virus; E, envelope; JEV, Japanese encephalitis virus; MERS-CoV, Middle East respiratory syndrome coronavirus; N, nucleocapsid; NS1, nonstructural 1; PLWH, persons living with HIV; PWOH, persons without HIV; RBD, receptor-binding domain; S1, subunit 1; S2, subunit 2; TBEV, tickborne encephalitis virus; YFV, yellow fever virus; WNV, West Nile virus; ZIKV, Zika virus.

Because most (83%) participants were PLHIV, we compared responses against coronavirus antigens to HIV-1–specific IgG responses in 173 samples (Figure 7, panels A, B). Participants from Thailand showed no IgG reactivity to HIV-1 antigens, reflecting the initiation of antiretroviral therapy in acute infection, typically before seroconversion; 34/38 participants received diagnoses by Fiebig stage III and initiated treatment immediately (Appendix Table 3). In contrast, most participants from Africa showed HIV-1 responses (median S/N 277), consistent with the initiation of antiretroviral therapy during chronic infection. However, higher HIV-1 responses for participants from Africa did not correlate with SARS-CoV-2 reactivity. Although S, S1, or S2 responses were higher in PLHIV than in persons without HIV-1, we noted little difference for RBD or N responses (Appendix Figure 7, panel A). In addition, we saw no correlation between coronavirus binding responses and HIV-1 markers of disease progression, either CD4+ T-cell counts or HIV-1 viral loads (Appendix Figure 7, panel B).

We also profiled responses against 23 flaviviruses and 1 alphavirus (Figure 7, panel C; Appendix Figure 8). Antibody responses did not show the dichotomous pattern seen between Thailand and Africa for coronavirus responses. Rather, flavivirus responses were seen in a subset of participants. Participant samples from Thailand often recognized most flavivirus antigens, typically with S/N >6. Among participants from Africa, samples from Nigeria and Uganda recognized several flavivirus antigens, but samples from Kenya and Tanzania seldom did. Some responses likely derived from yellow fever vaccination because we saw no comparable nonstructural 1 (NS1) protein responses. Responses might have been cross-reactive to common flavivirus epitopes because we often saw more responses to E than to NS1 antigens. We did not test binding responses to malaria antigens, but we had results of malaria smear tests for a subset of participants. Samples from 206 participants from Nigeria showed no difference in SARS-CoV-2 S2 IgG responses when comparing participants who had either a negative or positive malaria smear test (p = 0.15) (Appendix Figure 9). Together, these data demonstrate that higher reactivity among samples from Africa was not uniform across pathogens, emphasizing some genuinely higher coronavirus-like responses.

### SARS-CoV-2 Neutralization and Fc Effector Function

**Figure 8:**
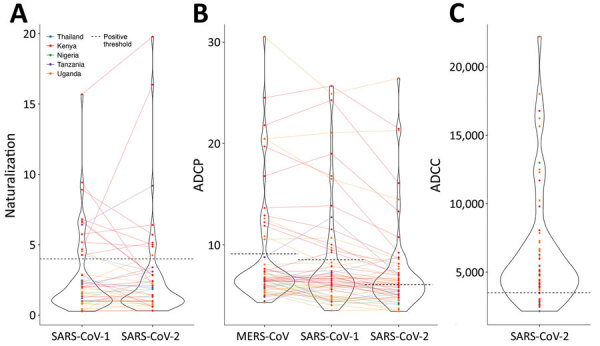
Violin plots of neutralizing, ADCP, and ADCC responses in prepandemic serums and plasma samples used to study coronavirus antibody responses before COVID-19 pandemic, Thailand and Africa. A) Pseudovirus neutralization against SARS-CoV-1 and SARS-CoV-2. The plot shows fold change of the ID_50_ for SARS-CoV-1 or SARS-CoV-2 over the ID_50_ for spike glycoprotein of the vesicular stomatitis virus control pseudoviruses. B) ADCP against MERS-CoV, SARS-CoV-1, and SARS-CoV-2. C) ADCC against SARS-CoV-2. Positive threshold is defined as mean of the negative control samples +3 SD. Solid lines link each sample between plots. Dotted lines indicate positive thresholds for each assay. Samples are color-coded for the participant’s country of origin. ADCC, antibody-dependent cellular cytotoxicity; ADCP, antibody-dependent cellular phagocytosis; ID_50_, 50% inhibitory dilution; MERS-CoV, Middle East respiratory syndrome coronavirus.

We tested for neutralization, ADCP, and ADCC in 60 samples (4 from Thailand, 21 from Kenya, 4 from Nigeria, 5 from Tanzania, and 26 from Uganda) with the highest outbreak coronavirus binding responses of the 173 samples with multiplex binding data. These samples represented the top 18 responders for IgG against N, RBD, and S against SARS-CoV-1 and SARS-CoV-2. Samples from 9 participants neutralized SARS-CoV-2; samples from 13 participants neutralized SARS-CoV-1 (Figure 8, panel A; Appendix Table 4). Most (8/9) samples that neutralized SARS-CoV-2 also neutralized SARS-CoV-1, and vice versa (8/13). Similarly, a subset of 30 participants showed strong ADCP against SARS-CoV-2, 15 against SARS-CoV-1, and 14 against MERS-CoV, and some samples had responses above the positive controls (Figure 8, panel B; Appendix Table 4). Most ADCP-positive samples showed responses against the 3 outbreak coronaviruses. For ADCC against SARS-CoV-2, most (48/60) participants showed responses above S/N >3 (Figure 8, panel C).

We found no strong relationship between binding and functional responses, even among samples with the most functionally relevant RBD responses or those recognizing multiple antigens, including antigens for RBD and N (Appendix Figure 10, panels A–C). We saw no correlation between neutralizing, ADCP, and ADCC responses (Appendix Figure 11). Functional responses were potent in a subset of participants, but these responses corresponded to a small fraction of the cohort: 0.4% for SARS-CoV-2 neutralization, 0.6% for SARS-CoV-1 neutralization, 1.3% for ADCP against SARS-CoV-2, 0.7% for ADCP against SARS-CoV-1, and 2.1% for ADCC against SARS-CoV-2.

## Discussion

We profiled antibody responses against 7 coronaviruses in a large multinational cohort of 2,250 persons from Thailand, Kenya, Nigeria, Tanzania, and Uganda, including PLHIV and persons without HIV-1. Among prepandemic samples, >5% had SARS-CoV-2–like responses to S or S2 antigens. We detected SARS-CoV-1 and MERS-CoV responses in a similar proportion of samples. We conducted our serosurvey in 2 steps: first, we screened for SARS-CoV-2 S2 reactivity; then, we selected reactive samples for further testing against 23 coronavirus antigens. We chose S2 because it is the most conserved segment of S across coronaviruses and sequence similarity for S2 between SARS-CoV-2 and the 6 other human coronaviruses ranges from 40% for 229E and NL63 to 91% for SARS-CoV-1; similarity for S1 ranges from 12% for 229E to 75% for SARS-CoV-1.

We observed less frequent responses to S-Trimer, RBD, or N compared with S, S1, or S2 responses, as previously reported (*4*,*7*,*8*,*10*,*19*). The limited S-Trimer, RBD, and N responses likely mark crucial gene functions like neutralization, whereas S or S2 responses could reflect the prevalence of cross-reactive responses, possibly linked to antibody-mediated Fc effector functions. We saw various antigen response combinations across participants, and we rarely saw persons with responses targeting all antigens from a given coronavirus. Furthermore, responses among outbreak coronaviruses correlated strongly and correlated with endemic coronavirus antigens; thus, we could not ascertain which pathogen or pathogens initiated the distinct recognition patterns across participants or whether specific responses are more functionally relevant.

We also characterized the neutralization, ADCP, and ADCC capacity of samples against outbreak coronaviruses. Some samples neutralized SARS-CoV-2, SARS-CoV-1, or both, but we saw no strong association between binding and neutralizing responses. Among 60 participants with the strongest binding responses to outbreak coronaviruses, ≈1/4 showed notable neutralization, ADCP, or ADCC responses. In the overall cohort, this number translates to <1% of participants, indicating that prior responses that could counteract SARS-CoV-2 infection were exceptionally rare in prepandemic samples. Nonetheless, some of these responses were high compared with responses induced after SARS-CoV-2 infection. What these functional responses signify and their clinical implications merit further clarification.

We showed that PLHIV had similar responses as HIV-negative persons, and PLHIV had even higher responses for some antigens. Rather than reflecting a true biologic difference, this finding likely is a statistical consequence of the higher percentage (83%) of PLHIV in the study. As such, we identified no association between coronavirus responses and typical markers of HIV-1 disease progression, such as viral loads and CD4+ T-cell counts. COVID-19 vaccine–induced immunity is less robust in PLHIV, especially for persons with low CD4+ T-cell counts or unsuppressed viremia (*20*–*25*), but our results indicate that this deficit is likely not linked to cross-reactive prepandemic responses.

Our most unexpected finding was that antibody responses against coronaviruses were much higher among participants from Africa than those from Thailand, especially for outbreak coronaviruses. No specific features distinguished participants from Africa and Thailand in our cohorts and we identified few differences across samples from the 4 countries in Africa. Previous studies showed differences across geographic settings, and higher SARS-CoV-2 antibody responses were detected in samples from sub-Saharan Africa than in samples from the United States (*19*). Because our knowledge of wild-type coronaviruses comes predominantly from Asia and SARS-CoV-1 spillover, we hypothesized that responses would be higher in Thailand. Although the mechanistic basis and functional consequences of more frequent responses in participants from Africa needs further study, our report underlines that our knowledge of the interplay between humans and coronavirus animal reservoirs remains vastly unexplored in Africa. Recent studies revealed that angiotensin-converting enzyme 2 (ACE2) use among bat coronavirus strains was not restricted to strains in Asia but was more broadly distributed; bat coronavirus RBD sequences from Bulgaria, Russia, and Kenya also used ACE2 (*26*–*30*). Further testing of animal reservoirs in Africa could elucidate whether additional bat coronavirus strains that readily use ACE2 are circulating.

To verify that high coronavirus responses seen in samples from Africa were specific, we tested 2 other antigen panels. The different reactivity profiles seen for coronavirus, HIV-1, or flavivirus antigens indicated that the SARS-CoV-2, SARS-CoV-1, and MERS-CoV responses observed among samples from Africa were not caused by high overall reactivity levels in the samples irrespective of the antigen and suggested that the responses could be coronavirus-specific. A previous report showed cross-reactivity between SARS-CoV-2 and Zika virus (*31*), but we saw no evidence of cross-reactivity against 8 Zika virus antigens tested, which aligns with another study (*32*). Multiple studies showed associations between SARS-CoV-2 antibody responses and malaria antigens (*11*,*33*–*36*). We did not test binding to malaria antigens but saw no difference in SARS-CoV-2 IgG responses between persons with positive or negative malaria smear tests. We investigated the possibility of SARS-CoV-2 cross-reactivity with flavivirus and HIV-1 antibody responses, but other pathogens could be the cause of the higher responses in participants from Africa than participants from Thailand. Nonetheless, the higher coronavirus-specific reactivity observed in samples from Africa warrants further analysis. Since the beginning of the pandemic, SARS-CoV-2 mortality rates have been lower in Africa than in other parts of the world. The younger population and underreporting of COVID-19 deaths likely contribute to this observation; nonetheless, hypothesizing that some preexisting coronavirus-specific responses affect COVID-19 disease severity is tempting. Further studies evaluating longitudinal samples obtained before and after the SARS-CoV-2 pandemic are needed to compare COVID-19 outcomes as a function of prepandemic cross-reactive coronavirus responses in Africa.

In conclusion, our study illustrates high coronavirus-specific reactivity in samples from Africa compared with samples from Thailand before the SARS-CoV-2 pandemic. Although we identified genuine antibody binding and neutralizing responses, such responses were rare, and their functional significance remains unclear. Findings from large coronavirus serosurveys can have implications for vaccine and monoclonal antibody distribution across global populations. Expanding such serosurveys to include diverse pathogens could help pandemic preparedness.

Appendix Additional information on coronavirus antibody responses before COVID-19 pandemic, Africa and Thailand.
