# Supplementary material for: Coronavirus Antibody Responses before COVID-19 Pandemic, Africa and Thailand
Source: Emerg Infect Dis. 2022 Nov;28(11):2214–25. doi: 10.3201/eid2811.221041 (PMC9622245; doi:10.3201/eid2811.221041)
Supplement: Appendix — Additional information on coronavirus antibody responses before COVID-19 pandemic, Africa and Thailand. [file 22-1041-Techapp-s1.pdf]

Article DOI: https://doi.org/10.3201/eid2811.221041

Coronavirus Antibody Responses before COVID-19 Pandemic, Africa and Thailand

Appendix

Antigen Panels

Some flavivirus antigens were produced in-house, as previously reported (*1*). Viral antigens were divided into 3 custom panels (Appendix Table 1). The coronavirus panel included 23 antigens from SARS-CoV, SARS-CoV-2, MERS-CoV, and 4 endemic hCoVs (229E, NL63, HKU1, and OC43) and included the spike (S) extra-cellular domain, subunit 1 (S1) and subunit 2 (S2), the nucleocapsid protein (N), the S receptor binding domain (RBD), and S-Trimer. The HIV-1 panel included 4 envelope antigens (gp120 and gp140). The flavivirus panel included 24 antigens corresponding to envelope (E) and nonstructural 1 (NS1) proteins from different flaviviruses (Zika virus, Dengue viruses 1–4, yellow fever virus, West Nile virus, Japanese encephalitis virus, and tickborne encephalitis virus) and to E1 from the alphavirus Chikungunya. As a control, each panel also included a human IgG.

References

1. Merbah M, Wollen-Roberts S, Shubin Z, Li Y, Bai H, Dussupt V, et al. A high-throughput multiplex assay to characterize flavivirus-specific immunoglobulins. J Immunol Methods. 2020;487:112874. PubMed https://doi.org/10.1016/j.jim.2020.112874

Appendix Table 1. Antigens, manufacturer, and expression system in antigen panels used to assess coronavirus antibody responses before COVID-19 pandemic, Africa and Thailand*

Antigen	Manufacturer†	Catalog no.	Expression system
SARS.2_S1	Sino Biological	40591-V08B1	Baculovirus-insect cells
SARS.1_S1	Sino Biological	40150-V08B1	Baculovirus-insect cells
MERS_S1	Sino Biological	40069-V08B1	Baculovirus-insect cells
SARS.2_RBD	LakePharma	46438	CHO-mammalian cells
SARS.1_RBD	Cambridge Biologics	01–03–0003	HEK293-human cells
MERS_RBD	Cambridge Biologics	01–03–0008	HEK293-human cells
SARS.2_S2	Sino Biological	40590-V08B	Baculovirus-insect cells
MERS_S2	Sino Biological	40070-V08B	Baculovirus-insect cells
SARS.2_Spike.Ecd	Sino Biological	40589-V08B1	Baculovirus-insect cells
SARS.2_Spike.Tri	LakePharma	46328	CHO-mammalian cells
SARS.1_Spike	BEI Resources	NR-722	Baculovirus-insect cells
MERS_Spike	Sino Biological	40069-V08B	Baculovirus-insect cells
229E_Spike	Sino Biological	40605-V08B	Baculovirus-insect cells
NL63_Spike	Sino Biological	40604-V08B	Baculovirus-insect cells
HKU1_Spike	Sino Biological	40606-V08B	Baculovirus-insect cells
OC43_Spike	Sino Biological	40607-V08B	Baculovirus-insect cells
SARS.2_N	Sino Biological	40588-V08B	Baculovirus-insect cells
SARS.1_N	Sino Biological	40143-V08B	Baculovirus-insect cells
MERS_N	Sino Biological	40068-V08B	Baculovirus-insect cells
229E_N	Sino Biological	40640-V07E	*Escherichia coli*
NL63_N	Sino Biological	40641-V07E	*E. coli*
HKU1_N	Sino Biological	40642-V07E	*E. coli*
OC43_N	Sino Biological	40643-V07E	*E. coli*
AntiHuman_IgG	SouthernBiotech	9042–01	Mouse
HIV.1_conM_GP120	HIV Reagent Program	12576	HEK293-human cells
HIV.1_AE.A244_GP120	HIV Reagent Program	12569	HEK293-human cells
HIV.1_C.1086_GP120	HIV Reagent Program	12582	HEK293-human cells
HIV.1_AG.ConCRF02_GP140	Produced in-house	NA	NA
ZIKV_PRV.WT_E.dom12	Produced in-house	NA	NA
ZIKV_PRV.WT_E.dom3	Produced in-house	NA	NA
ZIKV_Sur_NS1	The Native Antigen Company	ZIKVSU-NS1–100	HEK293-human cells
ZIKV_FP13_NS1	R&D Systems	9450-ZK-100	HEK293-human cells
ZIKV_Sur_E	The Native Antigen Company	ZIKVSU-ENV-100	HEK293-human cells
ZIKV_PRV.WT_E	Produced in-house	NA	NA
ZIKV_PRV.FL4_E	Produced in-house	NA	NA
ZIKV_ConAf_E	Produced in-house	NA	NA
JEV_SA14_E	Produced in-house	NA	NA
JEV_SA14_NS1	The Native Antigen Company	JEV-NS1–100	HEK293-human cells
YFV_17D_E	Produced in-house	NA	NA
YFV_17D_NS1	The Native Antigen Company	YFV-NS1–100	HEK293-human cells
DENV.4_Dom81_NS1	The Native Antigen Company	DENV4-NS1–100	HEK293-human cells
DENV.4_Dom81_E	The Native Antigen Company	DENV4-ENV-100	HEK293-human cells
DENV.3_PR98_NS1	Cal Bioreagents	A256	Baculovirus-insect cells
DENV.3_PR98_E	Cal Bioreagents	A252	Baculovirus-insect cells
DENV.2_Ind01_NS1	Cal Bioreagents	A255	Baculovirus-insect cells
DENV.2_Ind01_E	Cal Bioreagents	A251	Baculovirus-insect cells
DENV.1_VN07_NS1	Cal Bioreagents	A254	Baculovirus-insect cells
DENV.1_VN07_E	Cal Bioreagents	A250	Baculovirus-insect cells
WNV_NY99_E	The Native Antigen Company	REC31614–100	HEK293-human cells
WNV_NY99_NS1	The Native Antigen Company	WNV-NS1–100	HEK293-human cells
TBEV_Neu_NS1	The Native Antigen Company	TBEV-NS1–100	HEK293-human cells
CHIKV_NA_E1	The Native Antigen Company	CHIKV-E1–100	HEK293-human cells
Anti-human IgG	SouthernBiotech	9042–019042–01	Mouse

*CHIKV, chikungunya virus; DENV, dengue virus; JEV, Japanese encephalitis virus; MERS, Middle East respiratory syndrome; SARS, severe acute respiratory syndrome; TBEV, tickborne encephalitis virus; YFV, yellow fever virus; WNV, West Nile virus; ZIKV, Zika virus.
†BEI Resources, https://www.beiresources.org; Cal Bioreagents, http://www.calbioreagents.com; Cambridge Biologics, https://cambridgebiologics.com; HIV Reagent Program, https://www.hivreagentprogram.org; LakePharma, https://lakepharma.com; The Native Antigen Company, https://thenativeantigencompany.com; R&D Systems, https://www.rndsystems.com; Sino Biological, https://www.sinobiological.com; SouthernBiotech, https://www.southernbiotech.com.

Appendix Table 2. Demographic characteristics of study participants in a study of coronavirus antibody responses before COVID-19 pandemic, Africa and Thailand*

				Sex, %	
Protocol no.	Country	No. participants (% living with HIV)	Median age (range)	M	F
RV254	Thailand	598 (100)	30 (18–74)	98	2
RV466	Nigeria	206 (18)	34 (18–78)	55	45
RV329	Kenya	653 (86)	40 (19–77)	43	57
	Nigeria	160 (91)	38 (20–67)	42	58
	Tanzania	234 (89)	39 (19–74)	42	58
	Uganda	399 (79)	40 (20–70)	42	58

*We used serum samples collected from participants who were enrolled in ongoing studies of persons living with HIV and without HIV in Africa and Thailand. Samples were collected from 2013 through early 2020.

Appendix Table 3. Fiebig stage of 38 participants from Thailand in a study of coronavirus antibody responses before COVID-19 pandemic, Africa and Thailand*

Protocol no.	Country	Fiebig stage	No. (%)
RV254	Thailand	I	7 (18.42)
		II	7 (18.42)
		III	20 (52.63)
		IV	4 (10.53)

*We used serum samples collected from participants who were enrolled in ongoing studies of persons living with HIV and without HIV in Africa and Thailand. Samples were collected from 2013 through early 2020.

Appendix Table 4. Functional responses in samples from the 60 participants with the highest SARS-CoV-2 and SARS-CoV-1 binding responses in a study of coronavirus antibody responses before COVID-19 pandemic, Africa and Thailand*

Assay	Virus	Kenya, n = 21	Nigeria, n = 4	Tanzania, n = 5	Uganda, n = 26	Thailand, n = 4
Neutralization	SARS-CoV-1	10	0	1	1	1
Neutralization	SARS-CoV-2	7	0	1	1	0
ADCP	MERS-CoV	8	0	0	6	0
ADCP	SARS-CoV-1	9	0	1	5	0
ADCP	SARS-CoV-2	13	0	1	14	2
ADCC	SARS-CoV-2	14	4	5	25	0

*We used serum samples collected from participants who were enrolled in ongoing studies of persons living with HIV and without HIV in Africa and Thailand. Samples were collected from 2013 through early 2020. ADCC, antibody-dependent cellular cytotoxicity; ADCP, antibody-dependent cellular phagocytosis; MERS-CoV, Middle East respiratory syndrome coronavirus.

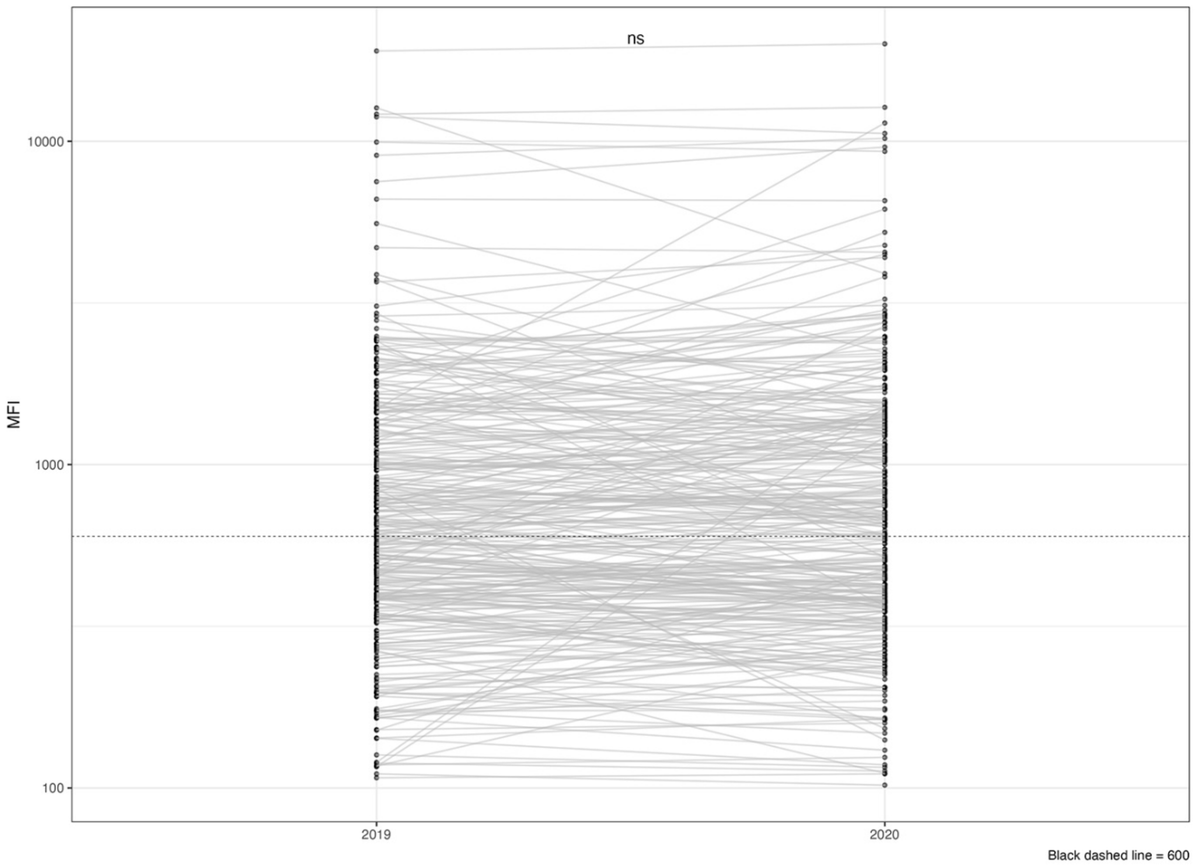

Appendix Figure 1. MFI for SARS-CoV-2 S2 responses in a study of coronavirus antibody responses before COVID-19 pandemic, Africa and Thailand. We compared IgG responses for 315 participants from Thailand with 2 samples collected an average of 4 months apart. The graph shows similar SARS-CoV-2 S2 responses in Thailand between 2019 and early 2020. Wilcoxon signed rank test showed no statistically significant difference between the 2 groups. MFI, mean fluorescence intensity; ns, no significance; S2, spike subunit 2 protein.

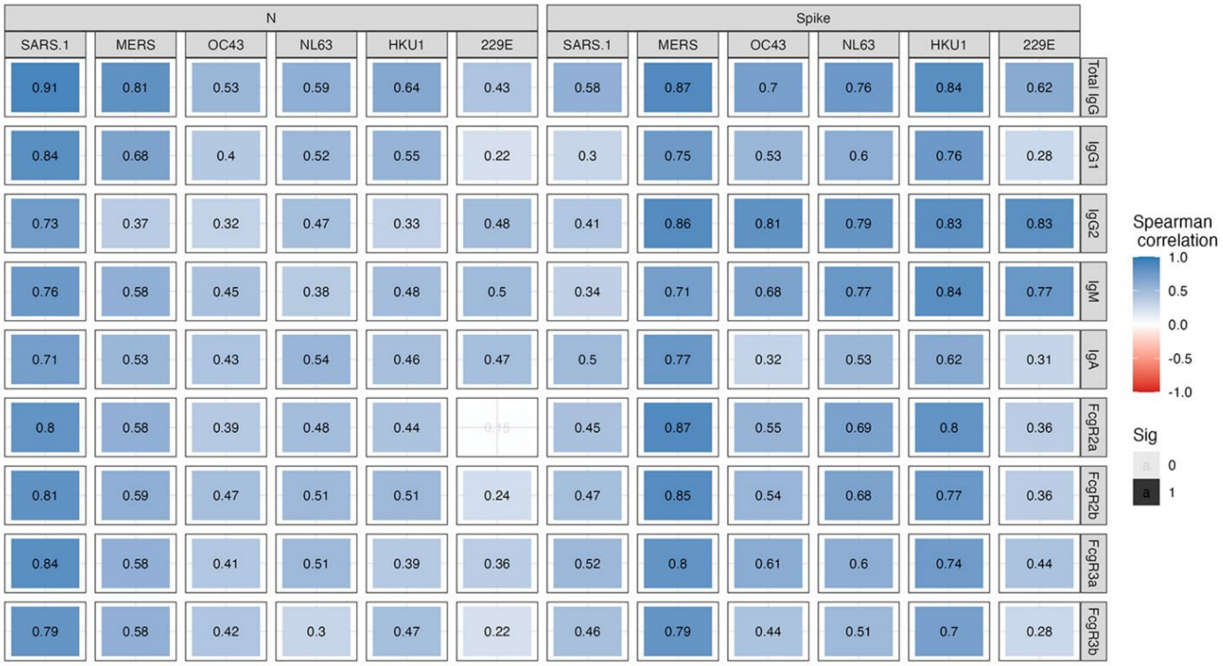

Appendix Figure 2. Correlation between binding responses against SARS-CoV-2 and 6 other coronaviruses in N and S proteins in a study of coronavirus antibody responses before COVID-19 pandemic, Africa and Thailand. We measured the relationship between N and S for SARS-CoV-2 and other coronaviruses across 173 samples by using Spearman's correlation, except for IgG3 and IgG4 due to low responses. Each square show's Spearman correlation values with p<0.05 after false discovery rate adjustment. FcgR, Fc gamma receptor (FcγRIIa, FcγRIIb, FcγRIIIa, and FcγRIIIb); N, nucleocapsid; S, spike; SARS.1, SARS-CoV-1; SARS.2, SARS-CoV-2; Sig, test significance.

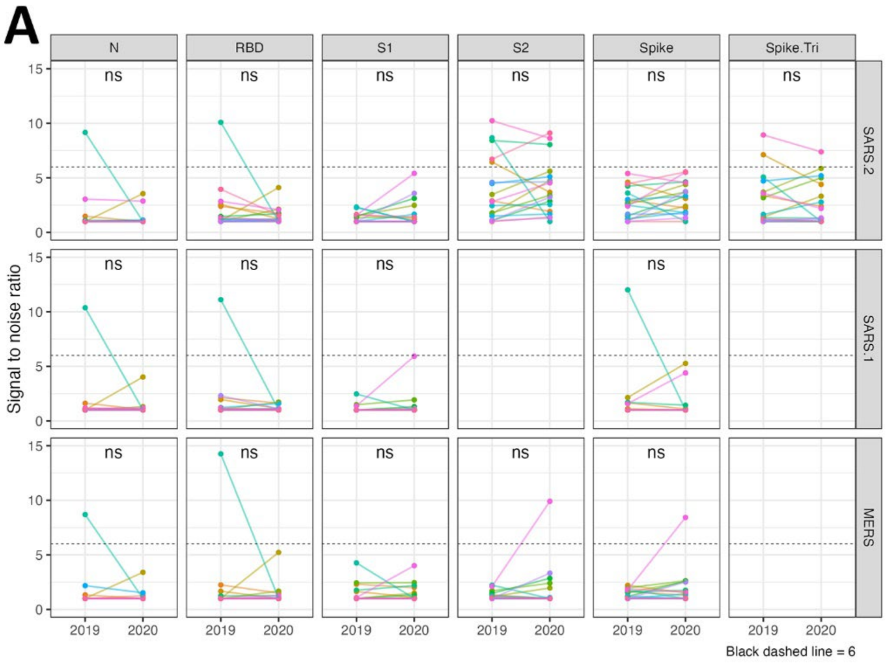

B

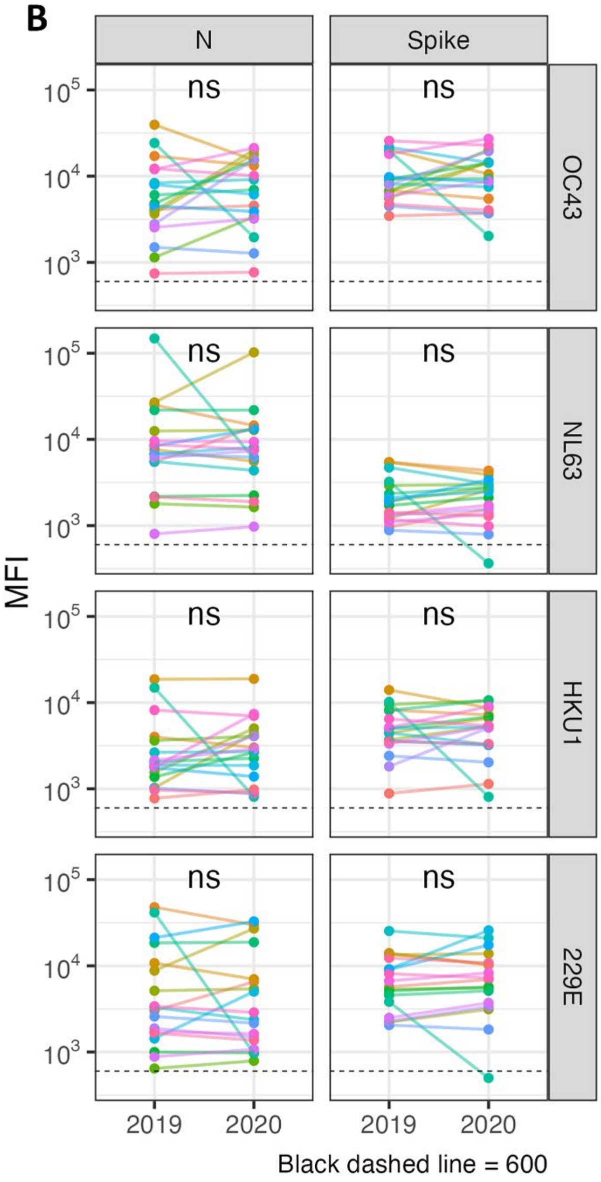

Appendix Figure 3. Coronavirus antigen reactivity in a study of coronavirus antibody responses before COVID-19 pandemic, Africa and Thailand. A) IgG responses to outbreak coronaviruses; B) IgG responses to 4 endemic coronaviruses. Results are from samples collected from participants in Thailand during August 2019–April 2020. Each color indicates 1 participant. We noted similar patterns across all coronaviruses and saw no difference in reactivity to coronavirus antigens between outbreak and endemic coronaviruses. The lack of significance of Wilcoxon signed rank tests is shown for each panel with ns (not significant) corresponding to p-values >0.05. MERS, Middle East respiratory syndrome coronavirus; MFI, mean fluorescence intensity; N, nucleocapsid; ns, no significance; RBD, receptor-binding domain; S, spike; S1, spike subunit 1; S2, spike subunit 2; SARS.1, SARS-CoV-1; SARS.2, SARS-CoV-2; Spike.Tri, S-Trimer.

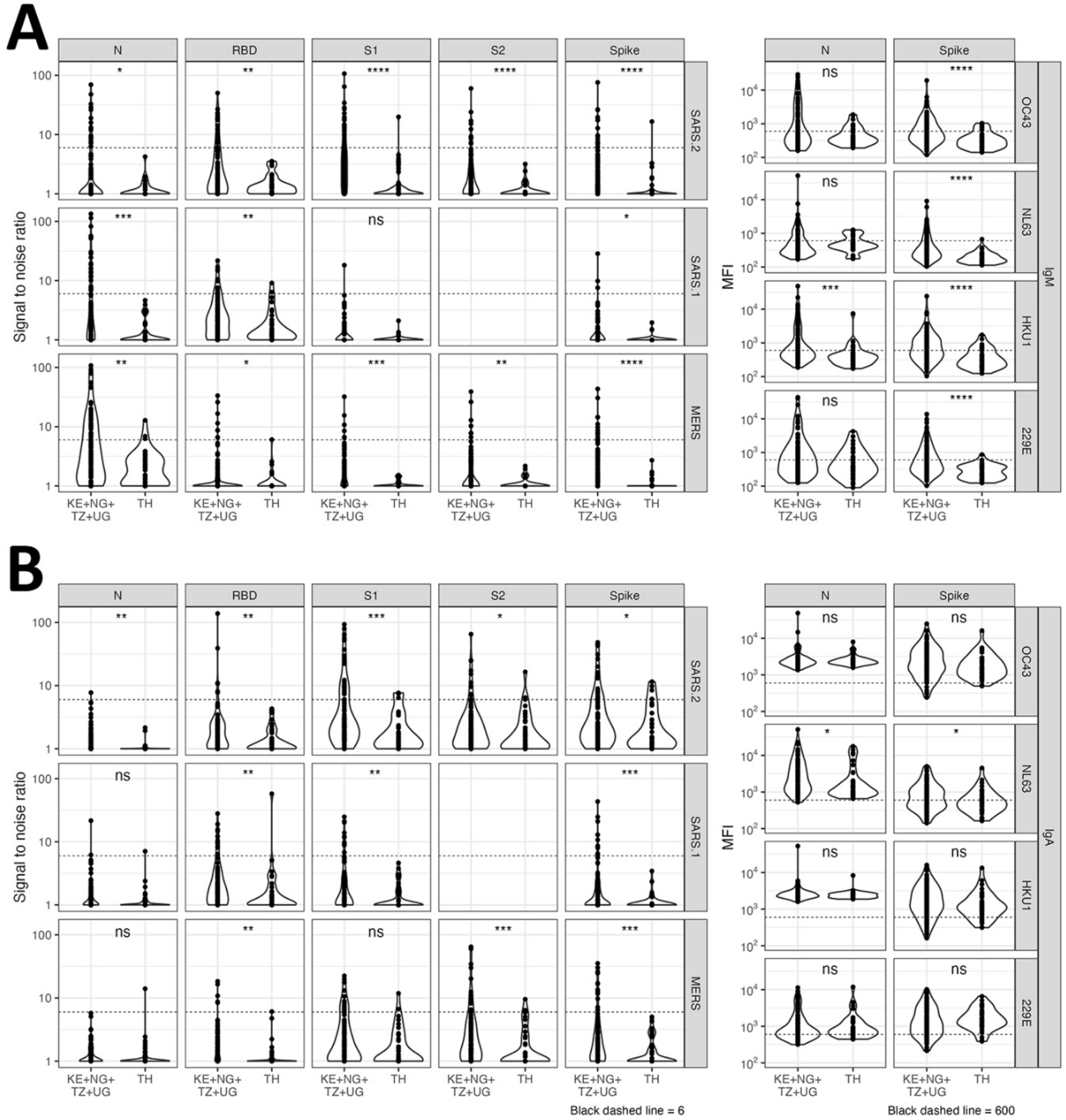

Appendix Figure 4. Violin plots of IgM and IgA coronavirus antibody responses before COVID-19 pandemic, Africa and Thailand. A) IgM responses; B) IgA responses. The figure shows responses across 14 antigens from 3 coronaviruses (left) and from 4 endemic coronaviruses (right) for samples from 117 participants from Africa and 38 from Thailand. We noted higher coronavirus responses in participants from Africa compared with participants from Thailand. We used Wilcoxon rank sum test to determine statistical significance; ns, p>0.05; *p≤0.05; **p≤0.01; ***p≤0.001; ****p≤0.0001. KE, Kenya; N, nucleocapsid; NG, Nigeria; ns, no significance; RBD, receptor-binding domain; S1, spike subunit 1; S2, spike subunit 2; SARS.1, SARS-CoV-1; SARS.2, SARS-CoV-2; TH, Thailand; TZ, Tanzania; UG, Uganda.

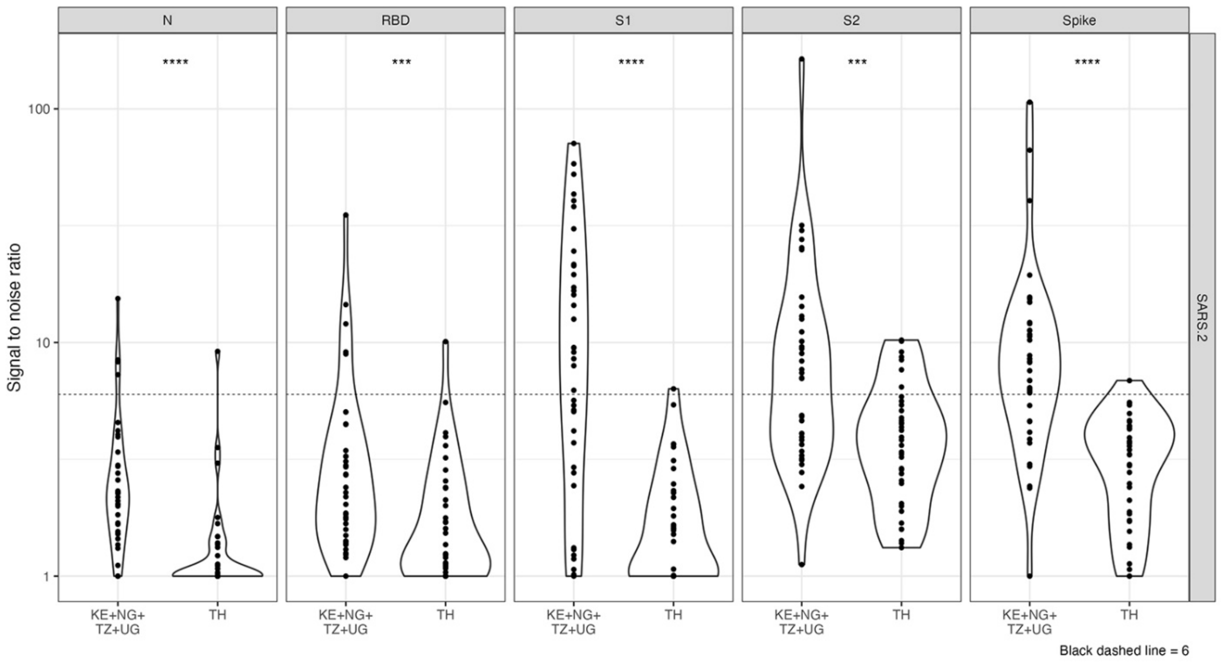

Appendix Figure 5. Violin plots showing SARS-CoV-2 responses in a study of pre–COVID-19 pandemic coronavirus antibody responses, Africa and Thailand. We downsampled data from countries in Africa to have the same sample size as Thailand (n = 38) to test whether higher coronavirus responses in Africa were due to the larger number of participants from Africa. We used Wilcoxon rank sum test to determine statistical significance; ns, p>0.05; *p≤0.05; **p≤0.01; ***p≤0.001; ****p≤0.0001. KE, Kenya; N, nucleocapsid; NG, Nigeria; ns, no significance; RBD, receptor-binding domain; S1, spike subunit 1; S2, spike subunit 2; SARS.2, SARS-CoV-2; TH, Thailand; TZ, Tanzania; UG, Uganda.

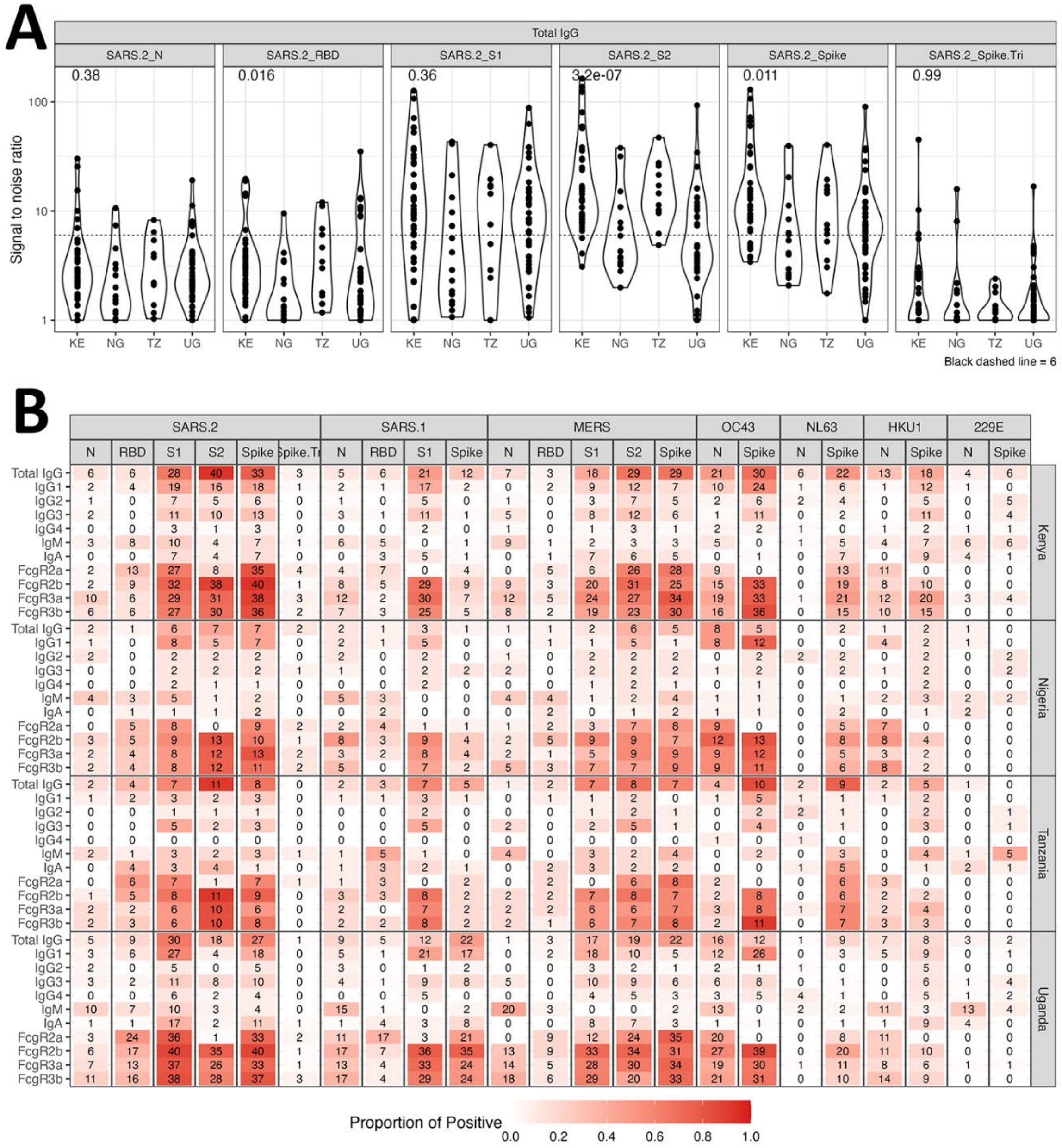

Appendix Figure 6. Coronavirus responses in a study of coronavirus antibody responses before COVID-19 pandemic, Africa and Thailand. A) Violin plots showing signal-to-noise ratio for IgG responses against 6 SARS-CoV-2 antigens. Numerals in each panel indicate p value for Kruskal-Wallis test. B) Number of positive samples using signal-to-noise ratio >6 across all coronavirus antigens and detection reagents. FcgR, Fc gamma receptor (FcγRIIa, FcγRIIb, FcγRIIIa, and FcγRIIIb); MERS, Middle East respiratory syndrome coronavirus; KE, Kenya; N, nucleocapsid; NG, Nigeria; RBD, receptor-binding domain; S1, spike subunit 1; S2, spike subunit 2; SARS.1, SARS-CoV-1; SARS.2, SARS-CoV-2; Spike.Tri, S-trimer; TH, Thailand; TZ, Tanzania; UG, Uganda.

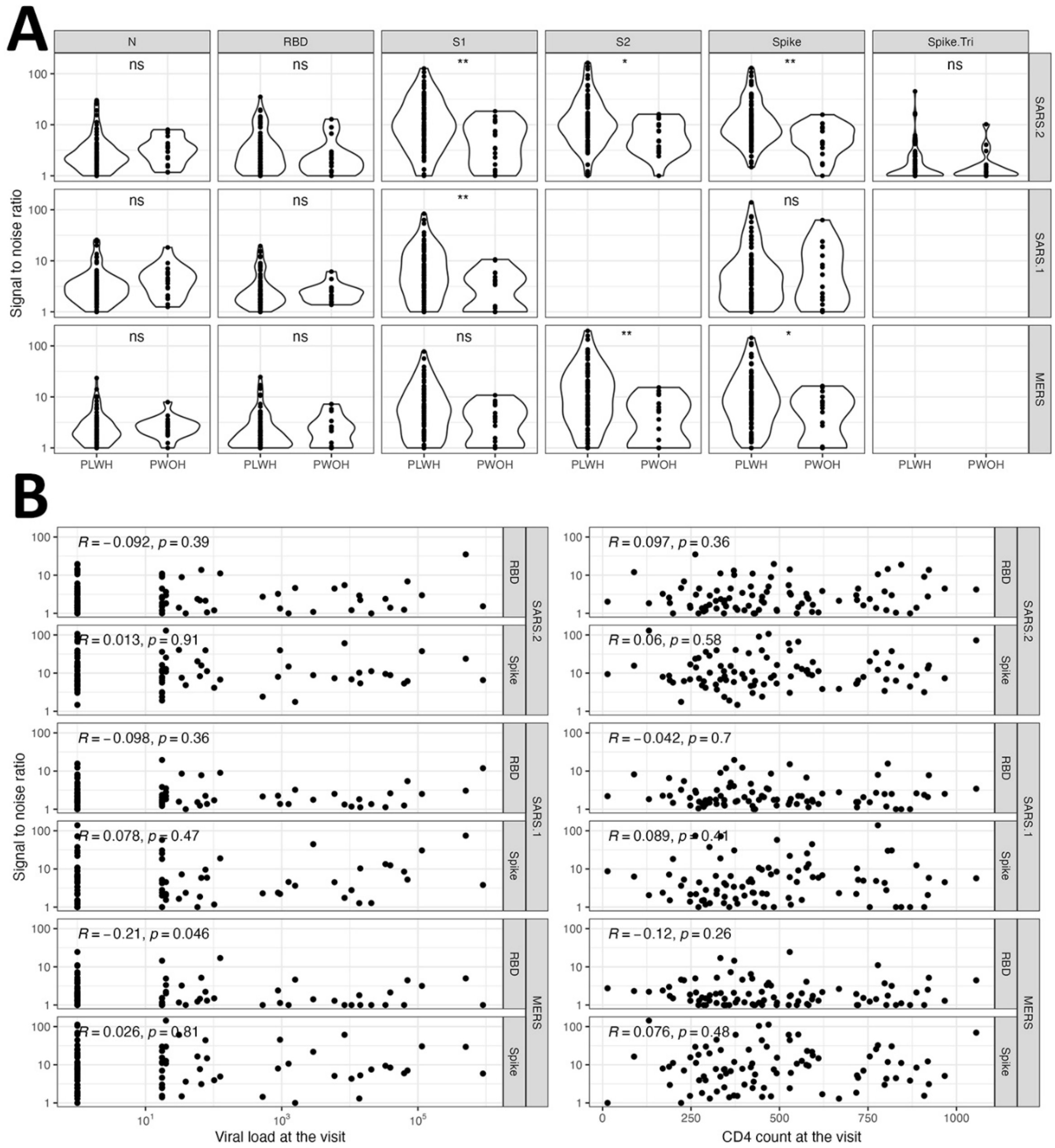

Appendix Figure 7. Correlation between coronavirus responses and HIV-1 status among participants from Africa in a study of coronavirus antibody responses before COVID-19 pandemic, Africa and Thailand. A) Violin plots showing comparison of IgG responses in persons with and without HIV-1. We used Wilcoxon rank sum test to determine statistical significance; ns, p>0.05; *p≤0.05; **p≤0.01. B) Scatterplots comparing IgG responses and HIV-1 viral loads (left) or CD4+ counts (right). Spearman ρ (*R*) and p values are shown in each panel. MERS, Middle East respiratory syndrome coronavirus; N, nucleocapsid; PLWH, persons living with HIV; PWOH, persons without HIV; RBD, receptor-binding domain; S1, spike subunit 1; S2, spike subunit 2; SARS.1, SARS-CoV-1; SARS.2, SARS-CoV-2; Spike.Tri, S-trimer.

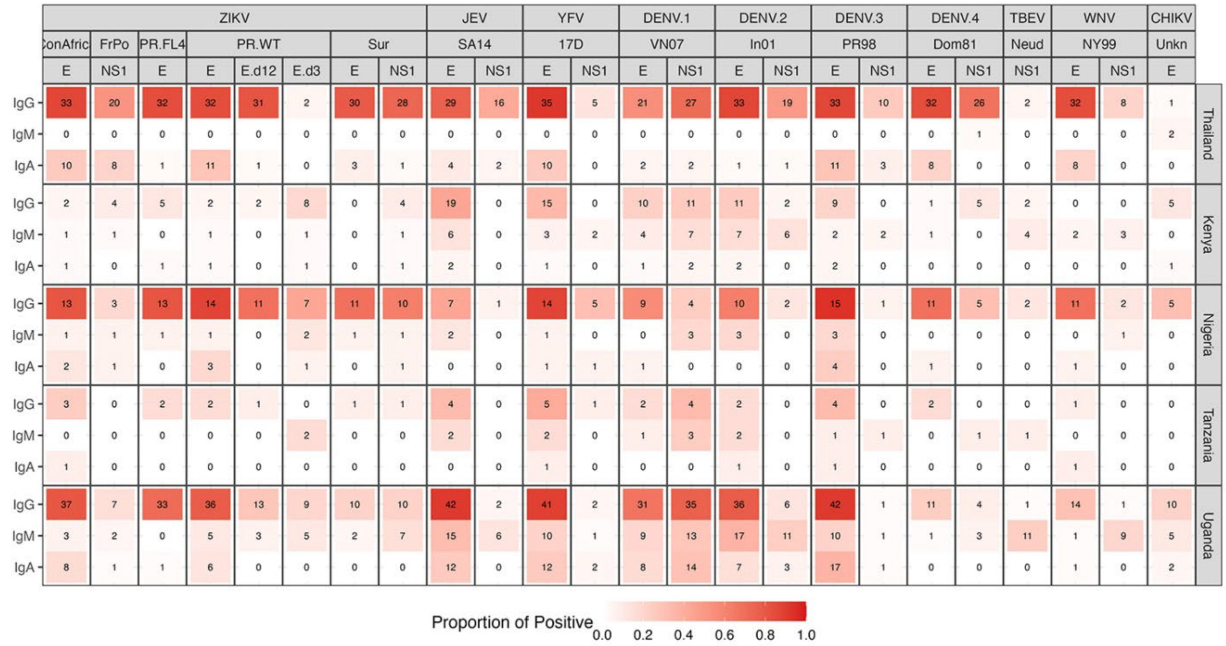

Appendix Figure 8. Proportion of samples positive for 23 flavivirus antigens and 1 alphavirus antigen in a study of coronavirus antibody responses before COVID-19 pandemic, Africa and Thailand. We used signal-to-noise ratio >6 to determine the number of positive samples across and all detections. CHIKV, chikungunya virus; DENV, dengue virus; E, envelope protein; JEV, Japanese encephalitis virus; NS1, nonstructural 1 protein; TBEV, tickborne encephalitis virus; YFV, yellow fever virus; WNV, West Nile virus; ZIKV, Zika virus

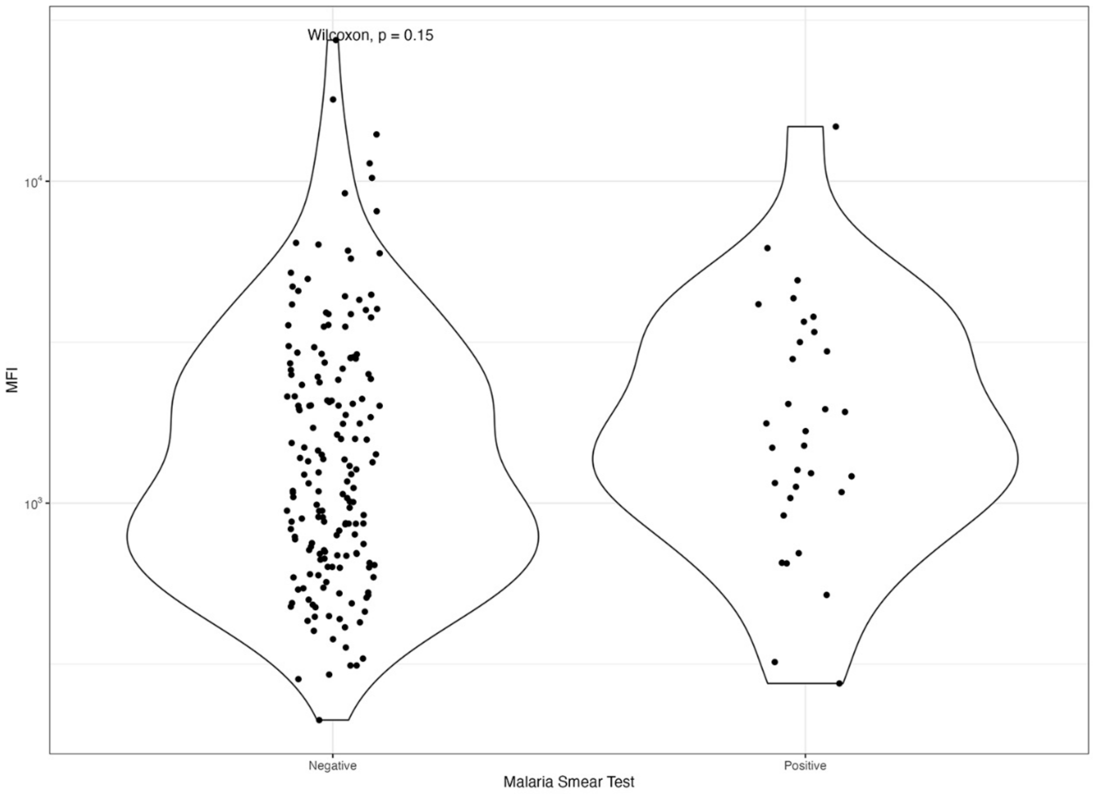

Appendix Figure 9. Violin plots of IgG responses against SARS-CoV-2 spike subunit 2 (S2) among participants also tested for malaria in a study of coronavirus antibody responses before COVID-19 pandemic, Africa and Thailand. Samples were from participants from Nigeria, 32 of whom had positive and 174 who had negative malaria blood smear tests. We noted similar IgG responses against SARS-CoV-2 S2 between the 2 groups and Wilcoxon rank sum test show no statistical difference ($p = 0.15$). Samples SARS-CoV-2 S2 IgG binding and malaria smear test were collected at the same time. MFI, mean fluorescence intensity.

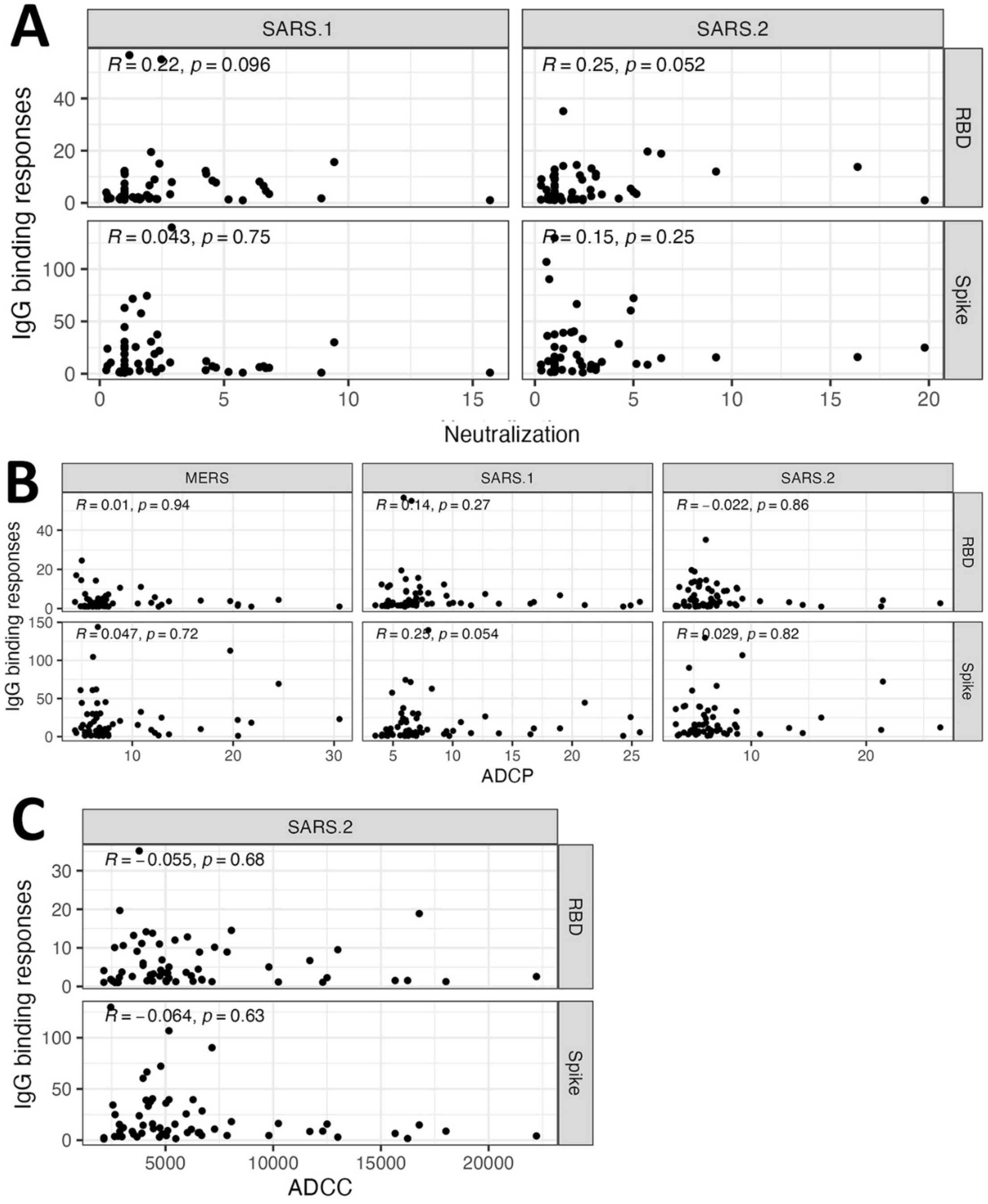

Appendix Figure 10. Relationship between binding, neutralization, ADCC, and ADCP responses in a study of coronavirus antibody responses before COVID-19 pandemic, Africa and Thailand. A) Relationship between binding and neutralization responses. B) Relationship between binding and ADCP responses. C) Relationship between binding and ADCC responses. Spearman ρ (*R*) and p values are shown in each panel. ADCC, antibody-dependent cellular cytotoxicity; ADCP, antibody-dependent cellular phagocytosis; MERS, Middle East respiratory syndrome coronavirus; RBD, receptor-binding domain; SARS.1, SARS-CoV-1; SARS.2, SARS-CoV-2.

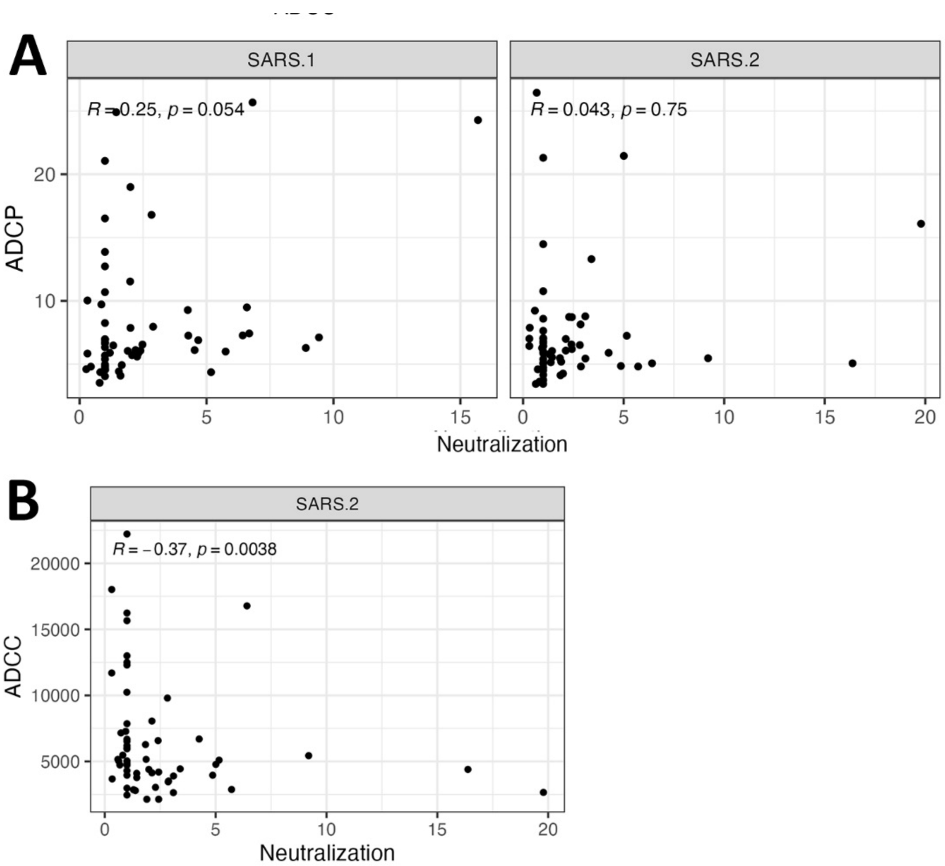

Appendix Figure 11. Relationship between neutralization, ADCC, and ADCP responses in a study of coronavirus antibody responses before COVID-19 pandemic, Africa and Thailand. A) Relationship between neutralization and ADCP responses. B) Relationship between neutralization and ADCC responses. Spearman ρ (*R*) and p values are shown in each panel. ADCC, antibody-dependent cellular cytotoxicity; ADCP, antibody-dependent cellular phagocytosis.
